# The association of leptin and C-reactive protein with the cardiovascular risk factors and metabolic syndrome score in Taiwanese adults

**DOI:** 10.1186/1475-2840-11-40

**Published:** 2012-04-25

**Authors:** Feng-Hsiang Chiu, Chung Hsun Chuang, Wen-Cheng Li, Yi-Ming Weng, Wen-Chih Fann, Hsiang-Yun Lo, Cheng Sun, Shih-Hao Wang

**Affiliations:** 1 Department of Emergency Medicine, Chang-Gung Memorial Hospital, Linkou Branch, No.5, Fu-Hsing Street, Guei-Shan, Taoyuan, Taiwan; 2 Department of Occupation Medicine, Chang-Gung Memorial Hospital, Keelung Branch, No. 222, Maijin Rd, Keelung, Taiwan; 3 Department of Emergency Medicine, Chang Gung Memorial Hospital, Chiayi Branch, No. 6, West sec. Chia-Pu Rd, Put-Zu, Chiayi, Taiwan; 4 Department of Emergency Medicine, Mackay Memorial Hospital, No. 92, Sec. 2, Zhongshan N. Rd., Zhongshan Dist, Taipei City, 10449, Taiwan; 5 Institute of Environmental and Occupational Health Science, National Yang- Ming University, Taipei, Taiwan; 6 Department of Occupation Medicine, Chang-Gung Memorial Hospital, No.5, Fu-Hsing Street, Guei-Shan, Taoyuan, Taiwan, 33305, R.O.C

**Keywords:** C-reactive protein, Leptin, Cardiovascular disease, Metabolic syndrome, Taiwan

## Abstract

**Background:**

Serum C-reactive protein (CRP) and leptin levels have been independently associated with the cardiovascular risk factors. The aim of the present study was to determine if their serum levels were associated with cardiovascular risk factors or metabolic syndrome as well as their correlation in the Taiwanese population.

**Methods:**

This retrospective study included 999 subjects (> 18 y), who underwent a physical examination in Chang-Gung Memorial Hospital-Linkou and Chiayi in Taiwan. The associations between CRP and/or leptin levels and cardiovascular risk factors and metabolic syndrome were determined using independent two sample t-tests to detect gender differences and chi-square tests to evaluate differences in frequencies. To compare the means of the variables measured among the four groups (high and low leptin and high and low CRP), analysis of variance (ANOVA) was used.

**Results:**

Both CRP and leptin levels were independently associated with several cardiovascular risk factors, including diabetes, hypercholesterolemia and metabolic syndrome in both men and women (*P* < 0.05). In addition, a positive correlation between leptin and CRP levels was observed in both genders. Both high-CRP and high-leptin were associated with high blood glucose, waist circumference and serum triglyceride. Whereas increased metabolic syndrome incidence was observed in males with elevated leptin regardless of CRP levels, females with elevated CRP or leptin had increased incidence of metabolic syndrome.

**Conclusion:**

Both leptin and CRP levels were associated with cardiovascular risk factors as well as metabolic syndrome score in both men and women although gender-specific differences were observed. Thus, CRP and leptin may represent useful biomarkers for predicting the onset of cardiovascular disease or metabolic syndrome in Taiwanese adults.

**Trial registration:**

IRB/CGMH 100-3514B

## Background

Because a combination of several cardiovascular and diabetes risk factors comprise metabolic syndrome, it has been postulated that factors involved in both diseases may interact. Two such factors include leptin and C-reactive protein (CRP), and both are associated with indices of obesity and cardiovascular disease.

CRP is an inflammatory marker of cardiovascular disease [1]; elevated levels are associated with increased risk of future coronary heart disease [2-4]. Obesity is one of the strongest determinants of CRP levels [5]. Furthermore, CRP has been related to metabolic syndrome in several studies [2,6], and its production is influenced by leptin [7].

Leptin is expressed by adipose tissue [8], regulating appetite and energy expenditure as well as insulin homeostasis [9]. Leptin levels predict metabolic syndrome development independent of obesity [10]. In men [11] and patients with type 2 diabetes [12], leptin levels were associated with cardiovascular disease. This association may be through inflammatory mechanisms since leptin and CRP levels have been correlated in a variety of patient populations [13-17].

In addition to cardiovascular disease, CRP and leptin levels are predictive of metabolic syndrome development. In middle-aged subjects, high leptin in men and high CRP in women were significant predictors of metabolic syndrome; those with elevations in both markers had the highest risk of developing metabolic syndrome [18]. However, the effects of CRP and leptin levels in a Taiwanese population have yet to be determined.

The objective of the present study was to determine if CRP and leptin levels are associated with cardiovascular disease risk factors as well as metabolic syndrome score in healthy Taiwanese adults. In addition, the possible correlation between CRP and leptin levels was also explored in this population. CRP and leptin may represent useful biomarkers for predicting the onset of cardiovascular disease or metabolic syndrome in Taiwanese adults.

## Methods

### Study population

In 2010, 1025 subjects (> 18 y), who had undergone a routine physical examination in Chang-Gung Memorial Hospital-Linkou (North Taiwan) and Chiayi (South Taiwan), were selected to participate in this retrospective study. Participants were included if they had undergone a physical exam in which the following parameters were assessed: weight, height, blood pressure, waist circumference, total cholesterol, triglyceride, fasting glucose, high density lipoprotein- cholesterol (HDL-C), low density lipoprotein-cholesterol (LDL-C), CRP, and leptin. Patients were exclude if they had not fasted for 12 h, were pregnant, or had a history of cardiovascular disease (e.g., myocardial infarction or stroke),chronic inflammatory disease (e.g., chronic rheumatoid arthritis, hepatitis, and cancer), or endocrine disease (e.g., hyperthyroidism or hypothyroidism). The study was approved by the Institute Reviewing Board of Chang Gung Memorial Hospital. As shown in Table 1, after 26 participants were excluded, a total of 999 participants were analyzed, including 720 males (35.79 ± 7.94 y) and 279 females (38.60 ± 12.80 y).

**Table 1:** Subject characteristics by gender

	Men (n = 720)	Women (n = 279)	*P*-value
Age (y)^1^	35.79 ± 7.94	38.60 ± 12.80	0.001
MS, n (%)^2^	61 (8.5)	59 (21.1)	<0.001
Diabetes mellitus, n (%)^2^	19 (2.6)	8 (2.9)	0.842
BMI (kg/m^2^)^1^	24.70 ± 3.43	21.90 ± 3.19	<0.001
Waist circumference (cm)^1^	81.76 ± 10.35	82.51 ± 9.67	0.294
Systolic BP (mm Hg)^1^	125.14 ± 13.27	113.08 ± 15.12	<0.001
Diastolic BP (mm Hg)^1^	78.69 ± 9.96	70.56 ± 9.83	<0.001
Fasting blood glucose (mg/dL)^1^	88.79 ± 12.69	90.09 ± 21.21	0.233
Total cholesterol (mg/dL)^1^	183.85 ± 32.84	184.53 ± 30.42	0.335
LDL cholesterol (mg/dL)^3^	119 (99, 139)	120 (99, 141)	0.855
HDL cholesterol (mg/dL)^3^	50 (43, 59)	50 (42, 59)	0.959
Triglycerides (mg/dL)^3^	91 (65, 128)	96 (68, 142)	0.119
Uric acid (mg/dL)^1^	6.28 ± 1.51	6.43 ± 1.50	0.163
Leptin (ng/mL)^3^	4.99 (3.03, 8.18)	5.51 (3.21, 9.56)	0.089
CRP (μg/mL)^3^	0.77 (0.42, 1.72)	0.85 (0.44, 1.79)	0.407

*P*-values are based on ^1^independent two sample *t*-test, ^2^Chi-square, or ^3^Mann-Whitney *U* test. Data are displayed as ^1^ mean ± SD, ^2^number (percentage), or ^3^ median (interquartile). Abbreviations: *MS,* metabolic syndrome; *BMI,* body mass index; *BP,* blood pressure; *LDL,* low density lipoprotein; *HDL,* high density lipoprotein; and *CRP,* C-reactive protein.

Participants were interviewed by trained nurses. Information regarding demographic (e.g., age, gender, etc.) and life style characteristics (e.g., history of smoking, drinking, etc.), history of illness and medication use, and physiological status (e.g., pregnancy, fasting time, etc.) was collected (Table 1).

### Blood pressure, BMI, and waist circumference measurements

Systolic and diastolic blood pressures were measured with a random zero sphygmomanometer while patients were in sitting position after a 5-min period of rest, according to the American Heart Association and Seventh Joint National Committee on Prevention, Detection, Evaluation, and Treatment of High Blood Pressure recommendations [19], and the average of up to three independent measurements was used.

To determine body mass index (BMI), the height and weight of each patient were measured using an automatic scale. BMI was calculated as weight (kg) divided by the height squared (m^2^).

Waist circumference was determined with the patient standing with their feet 25 to 30 cm apart. It was measured midway between the iliac crest and the lower margin of the 12th rib.

### Laboratory measurements

After a 12-h fast, venous blood samples were obtained between 5:30 to 11:00 a.m. and stored at 4°C. Total cholesterol, HDL-C, triglyceride (TG), fasting blood glucose (FBG), LDL-C, and leptin levels were determined for each participant. CRP was determined using a high sensitivity CRP assay.

### Cardiometabolic risk factor variables

Individuals were considered hypertensive if they were taking antihypertensive medications, self-reported a diagnosis of hypertension, if their systolic pressure was > 140 mmHg or diastolic pressure was > 90 mmHg, or if a combination of these features was recorded [20].

Participants were considered to have diabetes if they reported a current usage of antidiabetic medications, reported a previous diagnosis of diabetes, or had a FBG > 126 mg/dL [21].

A patient was considered to have dyslipidemia if they had the following: plasma TC ≥ 240 mg/dL and/or use of medications to lower blood cholesterol, TG ≥ 200 mg/dL, HDL-C < 40 mg/dL, and LDL-C ≥ 160 mg/dL and/or use of medications to lower LDL-C [22].

Regarding tobacco and alcohol use, those who had smoked ≥ 100 cigarettes in their lifetime were considered to have “ever smoked”. Alcohol use was defined as any alcohol use within the last year.

Leptin concentrations >4.99 ng/mL in men and >5.51 ng/mL in women were defined as “high”. CRP values >3 μg/mL in both men and women were defined as “high”.

### Definition of metabolic syndrome

A participant was diagnosed with metabolic syndrome if they presented with at least three of the following five factors: 1) high blood pressure (systolic blood pressure ≥ 130 mmHg and/or diastolic pressure ≥ 85 mmHg, under treatment, or current hypertension diagnosis); 2) high serum triglyceride (≥ 150 mg/dL or under treatment); 3) decreased HDL-C (< 40 mg/dL for males and < 50 mg/dL for females or under treatment); 4) hyperglycemia (FBG ≥ 100 mg/dL, under treatment, or diagnosis with diabetes mellitus); and 5) abdominal obesity [23]. Modified waist circumference cutoffs for Asian populations were used, which consisted of a waist circumference ≥ 90 cm for men and ≥ 80 cm for women [24].

### Statistical analysis

To compare the variables by gender or presence, independent two sample t-tests were used. For evaluating differences in frequencies, the chi-square test was used. Because leptin and CRP levels were not normally distributed, Mann–Whitney U tests were used.

To compare the means of the variables among the four groups (high and low leptin and high and low CRP), analysis of variance (ANOVA) was used. If the data was not normally distributed, a Kruskal-Wallis test was performed. When a significant difference between groups was apparent, multiple comparisons were performed using the Bonferroni procedure with type-I error adjustment. The correlation between leptin and CRP was determined using Spearman’s correlation coefficient. The partial correlations between metabolic syndrome components and CRP and leptin were calculated while controlling for age, or age and BMI. All statistical assessments were two-sided and considered significant when *P* < 0.05. Statistical analyses were performed using SPSS 15.0 statistical software (SPSS Inc, Chicago, IL).

## Results

### Participant characteristics

As shown in Table 1, the characteristics of the study subjects were compared by gender, and significant differences were observed. Specifically, women were significantly older and had a significantly lower BMI and systolic and diastolic blood pressures as compared to men (*P* ≤ 0.001). In addition, the prevalence of metabolic syndrome was significantly lower in male participants (8.5 vs. 21.1%, *P* < 0.001). No gender differences in leptin or CRP levels were observed.

### Associations between leptin and CRP levels with cardiovascular risk factors

Serum leptin levels were evaluated to determine if they were associated with cardiovascular risk factors in Taiwanese adults as previously reported in men [11] and those with type 2 diabetes [12]. In both men and women, diabetes, hypercholesterolemia and metabolic syndrome were significantly associated with elevated leptin levels (Table 2). In addition, gender differences were observed; men who were ever tobacco users, as well as those with hypertriglyceridemia and high LDL-C had significantly higher leptin levels. No such associations were detected in women.

**Table 2:** Leptin levels in patients with and without cardiovascular disease risk factors

	Men (n = 720)			Women (n = 279)		
	**Absent**	**Present**	***P*-value**	**Absent**	**Present**	***P*-value**
Diabetes	4.96 (2.97, 8.02)	6.97 (4.90, 8.56)	0.044	5.50 (3.14, 9.40)	8.65 (4.79, 12.35)	0.049
Hypercholesterolemia	4.94 (2.97, 7.85)	8.02 (4.56, 11.50)	0.003	5.43 (3.14, 9.40)	8.05 (5.67, 13.95)	0.039
Hypertriglyceridemia	4.91 (2.94, 7.89)	7.18 (4.44, 10.30)	0.002	5.39 (3.12, 9.22)	6.90 (4.90, 10.75)	0.054
High LDL cholesterol	4.92 (2.94, 7.85)	6.36 (3.73, 10.20)	0.013	5.43 (3.17, 9.87)	6.15 (4.45, 9.13)	0.561
Low HDL cholesterol	4.95 (2.94, 8.32)	5.04 (3.68, 7.43)	0.610	5.51 (3.12, 9.96)	5.51 (3.32, 9.13)	0.698
Hypertension	4.94 (2.97, 8.20)	5.51 (3.11, 7.93)	0.538	5.40 (3.12, 9.36)	7.67 (4.35, 11.20)	0.087
MS	4.78 (2.90, 7.73)	7.26 (4.98, 9.52)	<0.001	5.20 (2.94, 9.74)	6.63 (4.83, 9.40)	0.010
Ever smoked	4.82 (2.90, 7.60)	5.53 (3.35, 9.34)	0.030	5.51 (3.17, 9.56)	5.71 (4.57, 6.72)	0.829
Alcohol use	4.63 (2.89, 7.54)	5.25 (3.28, 8.57)	0.057	5.53 (3.32, 9.72)	5.40 (2.89, 9.40)	0.548

*P*-values are based on Mann–Whitney *U* test.

Data are displayed as median (interquartile).

Abbreviations: *LDL,* low density lipoprotein; *HDL,* high density lipoprotein; and *MS,* metabolic syndrome.

**Figure 1:**
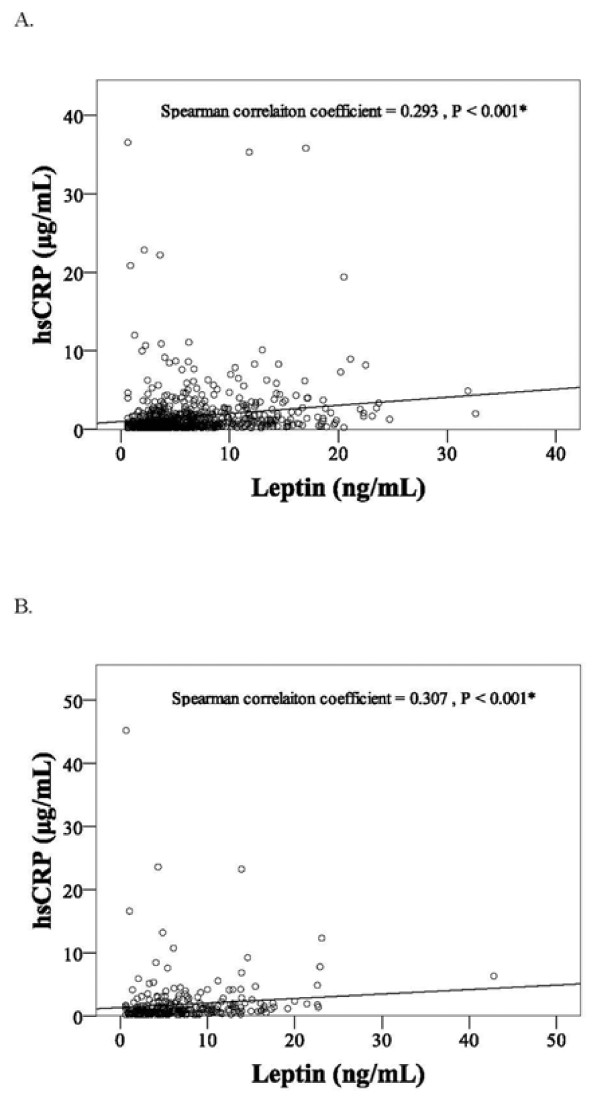
**The correlation between leptin and CRP.** The Spearman correlation coefficient between fasting plasma leptin and CRP was determined for both male (**A**) and female (**B**) participants. ^*^For male participants, the Spearman correlation coefficient = 0.293, *P* < 0.001. For female participants, spearman correlation coefficient = 0.307, *P* < 0.001.

In both genders, those with diabetes, hypercholesterolemia, hypertriglyceridemia, low HDL-C, and metabolic syndrome all had significantly higher CRP levels (Table 3). High LDL-C was associated with high CRP levels in men only. In addition, a significantly positive correlation between leptin and CRP levels was observed in both genders (*P* < 0.001, Figure 1).

**Table 3:** CRP levels in patients with and without cardiovascular disease risk factors

	Men (n = 720)			Women (n = 279)		
	**Absent**	**Present**	***P*-value**	**Absent**	**Present**	***P*-value**
Diabetes	0.76 (0.42, 1.67)	1.70 (0.55, 2.99)	0.044	0.82(0.43, 1.62)	2.42 (1.81, 4.06)	0.001
Hypercholesterolemia	0.75 (0.41, 1.67)	1.41 (0.81, 2.29)	0.002	0.82 (0.43, 1.64)	1.79 (1.15, 2.96)	0.013
Hypertriglyceridemia	0.74 (0.40, 1.65)	1.34 (0.76, 2.47)	<0.001	0.79 (0.39, 1.62)	1.46 (0.77, 2.09)	0.013
High LDL cholesterol	0.73 (0.40, 1.65)	1.14 (0.62, 2.04)	0.001	0.84 (0.40, 1.62)	1.21 (0.52, 2.76)	0.217
Low HDL cholesterol	0.72 (0.39, 1.53)	1.41 (0.67, 2.61)	<0.001	0.73 (0.39, 1.38)	1.06 (0.53, 2.14)	0.002
Hypertension	0.77 (0.41, 1.72)	0.78 (0.47, 1.68)	0.659	0.79 (0.42, 1.57)	1.56 (0.77, 3.72)	0.012
MS	0.72 (0.40, 1.55)	1.84 (0.87, 2.77)	<0.001	0.76 (0.39, 1.55)	1.33 (0.66, 2.81)	0.001
Ever smoked	0.76 (0.43, 1.70)	0.80 (0.40, 1.72)	0.291	0.83 (0.44, 1.69)	1.09 (0.85, 3.24)	0.279
Alcohol use	0.74 (0.41, 1.70)	0.80 (0.44, 1.72)	0.590	0.79 (0.42, 1.67)	1.05 (0.48, 2.00)	0.239

*P*-values are based on Mann–Whitney *U* test.

Data are displayed as median (interquartile).

Abbreviations: *CRP,* C-reactive protein; *LDL,* low density lipoprotein; *HDL,* high density lipoprotein; and *MS,* metabolic syndrome.

As shown in Table 4, male participants with high concentrations of both leptin and CRP had a significantly greater waist circumference, metabolic syndrome incidence, FBG, and triglyceride levels and lower HDL-C than individuals with low concentrations of both leptin and CRP (*P* < 0.017). Moreover, female participants with high concentrations of both leptin and CRP had significantly greater waist circumference, FBG, triglyceride and LDL-C levels than individuals with low leptin and CRP concentrations (*P* < 0.017, Table 4).

**Table 4:** Baseline characteristics by leptin and CRP level

	Men (n = 720)				Women (n = 279)			
**Variable**	^**a**^**Low leptin, low CRP (*n* = 335)**	**Low leptin, highCRP (*n* = 25**)	**High leptin, low CRP (*n* = 298)**	**High leptin, high CRP (*n* = 62)**	^**a**^**Low leptin, low CRP (*n* = 123)**	**Low leptin, high CRP (*n* = 16)**	**High leptin, low CRP (*n* = 119)**	**High leptin, high CRP (*n* = 21)**
Age (y)^1^	35.97 ± 7.76	35.76 ± 10.26	35.85 ± 8.02	34.58 ± 7.48	36.71 ± 13.65	42.56 ± 15.17	40.15 ± 11.73	37.90 ± 10.45
BMI (kg/m^2^)^1^	24.84 ± 3.55	24.22 ± 4.07	24.63 ± 3.38	24.49 ± 2.73	21.66 ± 3.13	22.03 ± 2.95	21.96 ± 3.23	22.90 ± 3.40
Waist circumference (cm)^1^	79.72 ± 7.90	78.17 ± 9.67	82.32 ± 11.42^b^	91.56 ± 10.99^b^	79.95 ± 7.77	84.54 ± 5.24	84.26 ± 10.62	86.04 ± 13.07^b^
MS, n (%)^2^	14 (4.2)	2 (8.0)	34 (11.4)^b^	11 (17.7)^b^	17 (13.8)	6 (37.5)^b^	31 (26.1)^b^	5 (23.8)
Ever smoked, n (%)^2^	98 (29.3)	7 (28.0)	101 (33.9)	24 (31.9)	4 (3.3)	1 (6.3)	3 (2.5)	2 (9.5)
Alcohol use, n (%)^2^	170 (50.7)	13 (52.0)	173 (58.1)	31 (50.0)	25 (20.3)	3 (18.8)	20 (16.8)	7 (33.3)
Systolic BP (mm Hg)^1^	124.86 ± 11.35	128.12 ± 24.54	125.89 ± 14.25	121.79 ± 11.21	111.02 ± 12.10	117.56 ± 15.60	113.81 ± 15.78	117.62 ± 23.81
Diastolic BP (mm Hg)^1^	78.32 ± 8.95	80.96 ± 16.48	79.27 ± 10.40	76.98 ± 9.53	69.06 ± 9.23	72.00 ± 9.65	71.32 ± 9.43	73.90 ± 14.05
Fasting blood glucose (mg/dL)^1^	87.66 ± 9.29	87.12 ± 6.950	88.51 ± 10.71	96.87 ± 27.80^b^	87.20 ± 8.06	103.13 ± 63.91^b^	89.25 ± 8.66	101.90 ± 44.87^b^
Total cholesterol (mg/dL)^1^	181.40 ± 29.75	172.64 ± 30.46	185.68 ± 35.19	192.79 ± 35.93	178.28 ± 27.46	189.63 ± 37.21	190.00 ± 31.80^b^	186.24 ± 28.59
Triglycerides (mg/dL)^3^	87 (64, 117)	84 (61, 99)	98 (64, 135)	125 (79, 153)^b^	86 (65, 125)	119 (73, 155)	101 (68, 159)	136 (83, 151)^b^
LDL cholesterol (mg/dL)^3^	118 (99, 138)	117 (99, 129)	120 (96, 139)	126 (112, 150)	113 (95, 133)	121 (110, 155)	124 (102, 144)	129 (109, 141)^b^
HDL cholesterol (mg/dL)^3^	51 (43, 59)	46 (40, 53)	51 (43, 60)	45 (40, 50)^b^	51 (44, 59)	42 (38, 50)^b^	51 (43, 61)	50 (40, 55)

^a^ The low leptin-low CRP group was considered the reference group.

^b^ indicates a significant difference between indicated group and the reference group,

*P*-values are based on ^1^ ANOVA test, ^2^ Chi-square, or ^3^ Kruskal-Wallis test.

Data are displayed as ^1^ mean ± SD, ^2^number (percentage), or ^3^ median (interquartile).

Abbreviations: *CRP,* C-reactive protein; *BMI,* body mass index; *MS,* metabolic syndrome; *BP,* blood pressure; *LDL,* low density lipoprotein; and *HDL,* high density lipoprotein.

### Associations between leptin and CRP levels with metabolic syndrome

**Figure 2:**
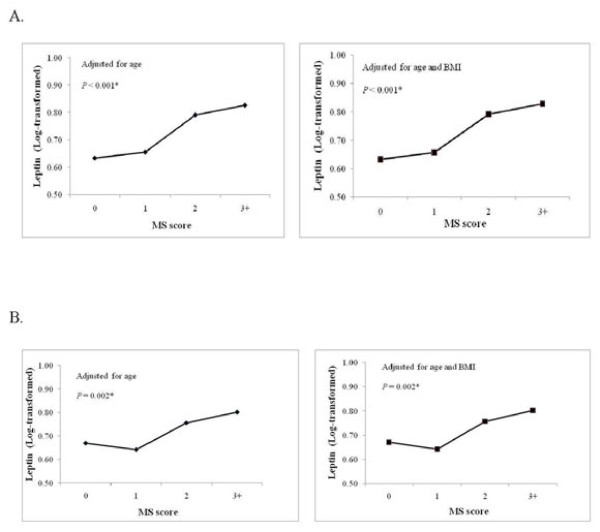
**Relationship between fasting plasma leptin concentrations and metabolic syndrome score.** The association between fasting plasma leptin concentration and metabolic syndrome score was determined for male (**A**) and female (**B**) participants and adjusted for either age (left panel) or age and BMI (right panel).

**Figure 3:**
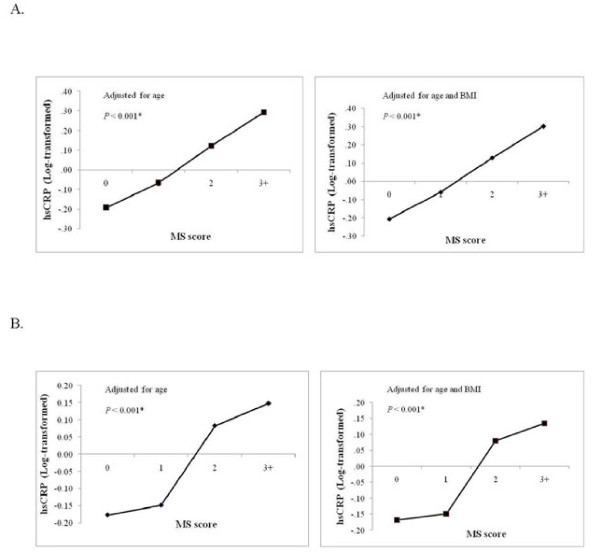
**Relationship between fasting plasma CRP levels and metabolic syndrome score.** The association between fasting plasma CRP level and metabolic syndrome score was determined for male (**A**) and female (**B**) participants and adjusted for either age (left panel) or age and BMI (right panel).

The associations between leptin and CRP levels with metabolic syndrome score, which is determined as the number of metabolic syndrome components described by the ATP III criteria [23], was next determined. Leptin concentrations varied in relation to the metabolic syndrome score in both genders (  2). After adjusting for age, leptin levels significantly increased with increasing metabolic syndrome score in both male and female subjects (Figure 2, left panels; *P* < 0.05). Upon additional adjustment for BMI, the association between elevated leptin levels with metabolic syndrome score remained in both male (*P* < 0.001) and female (*P* = 0.002) subjects (Figure 2, right panels). Similar associations were observed for CRP levels (Figure 3). After adjusting for age, CRP levels increased with increasing metabolic syndrome score in both male and female subjects (*P* < 0.001). Upon additional adjustment for BMI, the association of CRP with metabolic syndrome score remained significant in both male and female subjects (*P* < 0.001).

## Discussion

This is the first study to explore the possible association and interaction between leptin and CRP levels with risk factors for cardiovascular disease and metabolic syndrome in a Taiwanese population. Both leptin and CRP levels were associated with cardiovascular risk factors as well as metabolic syndrome score in both men and women as in Xu et al. [25] although gender-specific differences were observed. Specifically, significant correlations were observed between both leptin and CRP levels with diabetes, hypercholesterolemia, and metabolic syndrome; however, gender differences were identified regarding the association between these markers and the presence of individual cardiovascular disease risk factors. Furthermore, individuals with both high leptin and high CRP levels had significantly greater waist circumference, glucose levels, triglyceride levels as compared to those with low leptin and low CRP levels. Elevated leptin and low CRP levels were associated with metabolic syndrome in both men and women. Finally, a positive correlation between leptin and CRP levels was observed as compared to previous reports [17,18].

In a previous report, we observed that leptin levels were associated with cardiovascular disease risk factors [26]. In this study, further associations between leptin and CRP levels and metabolic syndrome score were detected in both male and female participants. This is different from a previous report by Ukkola et al. in which metabolic syndrome scores were associated with high leptin and high CRP in men and with high CRP only in women after stratification by BMI in a Finish population [18]. Thus, there may be population-specific differences in the correlation between BMI and leptin level, which will require further studies to elucidate.

The association between leptin and metabolic syndrome is not fully understood. Leptin receptors are found on pancreatic β cells, suppressing insulin secretion [27]. In leptin resistance, glucose-stimulated insulin secretion is not suppressed by leptin, which may lead to insulin resistance [28]. Further studies are required to determine the exact role of leptin in metabolic syndrome.

As previously reported in a Finnish population [17] as well as Japanese type 2 diabetic patients [13] and healthy individuals [29], an association between leptin and CRP was observed in the Taiwanese cohort analyzed in the present study. The mechanisms linking leptin with CRP are not well known. Among the proinflammatory cytokines, CRP synthesis is primarily regulated by interleukin-6 (IL-6) [30]. Proinflammatory cytokines, which include IL-6, IL-1 and tumor necrosis factor-alpha (TNF-α) are secreted not only by adipocytes but also by inflammatory cells, such as adipose-resident macrophages [31-33]. These cytokines may stimulate leptin secretion from human adipose tissue as well as hepatic CRP synthesis [34,35]. However, leptin itself can stimulate the production of various cytokines, including IL-6 [36]. Thus, IL-6 and leptin may positively regulate the other’s expression through a feedback loop. Because the leptin receptor mediates intracellular signaling with a specificity similar to IL-6-type receptors [37], leptin may regulate CRP production not only via IL-6 but also via the leptin receptor. In addition, Chen et al. [38] demonstrated that physiological concentrations of leptin can dose-dependently stimulate CRP expression in human primary hepatocytes. An alternative explanation is that obesity upregulates the production of both leptin and a cytokine regulating CRP synthesis without a causal relationship between leptin and CRP. However, further studies are necessary to determine the exact intracellular mechanisms by which leptin regulates CRP production. Furthermore, a role for CRP in the development of leptin resistance has also been suggested, but needs to be further analyzed [39,40].

In the present study, leptin and CRP levels were associated with metabolic syndrome score, indicating that high levels of these markers may be predictive of developing metabolic syndrome similarly to that shown for high baseline fasting insulin levels [41]. Further follow-up analysis will be undertaken to fully evaluate the predictive value of these markers using a prediction model as was used by Chien et al. [42].

Differences in cardiovascular risk have been observed in obese individuals with and without adipose tissue inflammation. In a recent study by Farb et al. [43], higher plasma insulin, triglyceride, glucose, blood pressure, LDL-C and high sensitivity CRP and lower HDL-C were observed in individuals with inflamed adipose. Further studies are necessary to determine if adipose inflammation alters the association of CRP and leptin with metabolic syndrome in obese individuals.

Limitations of this study include its retrospective, cross-sectional nature. In addition, the data used in the present study was collected during a routine physical examine. Because examination of CRP and leptin levels was by choice, this may select for individuals concerned about weight and/or metabolic problems. Furthermore, no information regarding exercise and diet as well as hereditary disease was collected.

## Conclusions

In conclusion, this study suggests that leptin and CRP are strong predictors of not only cardiovascular disease but also metabolic syndrome in Taiwanese men and women. The associations between leptin and CRP with cardiovascular disease risk factors and metabolic syndrome score may provide a tool for assessing the risk of developing these diseases although further longitudinal studies in larger patient populations are necessary to determine the predictive value of examining their levels.

## Abbreviations

ANOVA: Analysis of variance; BMI: Body mass index; CRP: C-reactive protein; FBG: Fasting blood glucose; HDL-C: High density lipoprotein- cholesterol; IL-6: Interleukin-6; LDL-C: Low density lipoprotein-cholesterol; TG: Triglyceride; TNF-α: Tumor necrosis factor-alpha.

## Competing interests

We declared that there is no conflict of interests in the submission and no funding support was received for the study.

## Authors’ contributions

WCL and YMW contributed to Study Design; FHC, CCH, HYL, CS and SHW contributed to Conduct/data collection; WCF contributed to Data Analysis; FHC and CCH contributed to Manuscript Writing. All authors read and approved the final manuscript.

## References

[B1] Jialal I Devaraj S Role of C-reactive protein in the assessment of cardiovascular risk Am J Cardiol 2003 91 200 202 10.1016/S0002-9149(02)03110-7 12521635

[B2] Ridker M Buring JE Cook NR Rifai N C-reactive protein, the metabolic syndrome, and risk of incident cardiovascular events: an 8-year follow-up of 14719 initially healthy American women Circulation 2003 107 391 397 10.1161/01.CIR.0000055014.62083.05 12551861

[B3] Koenig W Sund M Fröhlich M Fischer HG Löwel H Döring A Hutchinson WL Pepys MB C-reactive protein, a sensitive marker of inflammation, predicts future risk of coronary heart disease in initially healthy middle-aged men. Results from the MONICA (Monitoring Trends and Determinants in Cardiovascular Disease) Ausburg Cohort Study Circulation 1999 99 237 242 10.1161/01.CIR.99.2.237 9892589

[B4] Tracy RP Lemaitre RN Psaty BM Ives DG Evans RW Cushman M Meilahn EN Kuller LH Relationship of C-reactive protein to cardiovascular disease in the elderly: Results from the Cardiovascular Health Study and the Rural Health Promotion Project Arterioscler Thromb Vasc Biol 1997 17 1112 1121 10.1161/01.atv.17.6.1121 9194763

[B5] Visser M Bouter LM McQuillan GM Wener MH Harris TB Elevated C-reactive protein levels in overweight and obese adults JAMA 1999 282 2131 213 10.1001/jama.282.22.2131 10591334

[B6] Rutter MK Meigs JB Sullivan LM D’Agostino RB Wilson PW C-reactive protein, the metabolic syndrome, and prediction of cardiovascular events in the Framingham Offspring Study Circulation 2004 110 380 385 10.1161/01.CIR.0000136581.59584.0E 15262834

[B7] Dowidar NL Dejong CHC Fearon KCH Garden OJ Ross JA Effects of leptin on isolated human hepatocyte C reactive protein production Eur J Gastroenterol Hepatol 2000 12 A18

[B8] Mantzoros CS The role of leptin in human obesity and disease: a review of current evidence Ann Intern Med 1999 130 671 680 10215564 10.7326/0003-4819-130-8-199904200-00014

[B9] Halaas JL Gajiwala KS Maffei M Cohen SL Chait BT Rabinowitz D Lallone RL Burley SK Friedman JM Weight-reducing effects of the plasma protein encoded by the obese gene Science 1995 269 543 546 10.1126/science.7624777 7624777

[B10] Franks PW Brage S Luan J Ekelund U Rahman M Farooqi IS Halsall I O’Rahilly S Wareham NJ Leptin predicts a worsening of the features of the metabolic syndrome independently of obesity Obes Res 2005 13 1476 1484 10.1038/oby.2005.178 16129731

[B11] Wallace AM McMahon AD Packard CJ Kelly A Shepherd J Gaw A Sattar N Plasma leptin and the risk of cardiovascular disease in the west of Scotland coronary prevention study (WOSCOPS) Circulation 2001 104 3052 3056 10.1161/hc5001.101061 11748099

[B12] Trayhurn P Wood IS Adipokines: inflammation and the pleiotropic role of white adipose tissue Br J Nutr 2004 92 347 355 10.1079/BJN20041213 15469638

[B13] Yanagawa T Taniguchi A Fukushima M Nakai Y Nagasaka S Ohgushi M Matsumotoe K Kuroea A Ohyaa M Seinoa Y Leptin, triglycerides, and interleukin 6 are independently associated with C-reactive protein in Japanese type 2 diabetic patients Diabetes Res Clin Pract 2007 75 2 6 10.1016/j.diabres.2006.04.019 16764962

[B14] Kazumi T Kawaguchi A Hirano T Yoshino G C-reactive protein in young, apparently healthy men: associations with serum leptin, QTc interval, and high-density lipoprotein-cholesterol Metabolism 2003 52 1113 1116 10.1016/S0026-0495(03)00184-7 14506615

[B15] Shamsuzzaman AS Winnicki M Wolk R Svatikova A Phillips BG Davison DE Berger PB Somers VK Independent association between plasma leptin and C-reactive protein in healthy humans Circulation 2004 109 2181 2185 10.1161/01.CIR.0000127960.28627.75 15117839

[B16] Ble A Windham BG Bandinelli S Taub DD Volpato S Bartali B Tracy RP Guralnik JM Ferrucci L Relation of plasma leptin to C-reactive protein in older adults (from the Invecchiare nel Chianti study) Am J Cardiol 2005 96 991 995 10.1016/j.amjcard.2005.05.058 16188530

[B17] Viikari LA Huupponen RK Viikari JS Marniemi J Eklund C Hurme M Lhetimaki T Kivimaki M Raitakari OT Relationship between leptin and C-reactive protein in young Finnish adults J Clin Endocrinol Metab 2007 92 4753 4758 10.1210/jc.2007-0103 17878255

[B18] Ukkola O Kesäniemi YA Leptin and high-sensitivity C-reactive protein and their interaction in the metabolic syndrome in middle-aged subjects Metabolism 2007 56 1221 1227 10.1016/j.metabol.2007.04.019 17697865

[B19] Chobanian AV Bakris GL Black HR Cushman WC Green LA Izzo JL Jr Jones DW Materson BJ Oparil S Wright JT Jr Roccella EJ Seventh report of the Joint National Committee on Prevention, Detection, Evaluation, and Treatment of High Blood Pressure Hypertension 2003 42 1206 1252 10.1161/01.HYP.0000107251.49515.c2 14656957

[B20] Chobanian AV Bakris GL Black HR Cushman WC Green LA Izzo JL Jr Jones DW Materson BJ Oparil S Wright JT Jr Roccella EJ National Heart, Lung, and Blood Institute Joint National Committee on Prevention, Detection, Evaluation, and Treatment of High Blood Pressure; National High Blood Pressure Education Program Coordinating Committee: The Seventh Report of the Joint National Committee on Prevention, Detection, Evaluation, and Treatment of High Blood Pressure: The JNC 7 Report JAMA 2003 289 2560 2572 10.1001/jama.289.19.2560 12748199

[B21] Report of the Expert Committee on the Diagnosis and Classification of Diabetes Mellitus Diabetes Care 1997 20 1183 1197 9203460 10.2337/diacare.20.7.1183

[B22] National Cholesterol Education Program (NCEP) Expert Panel on Detection, Evaluation, and Treatment of High Blood Cholesterol in Adults (Adult Treatment Panel III) Third Report of the National Cholesterol Education Program (NCEP) Expert Panel on Detection, Evaluation, and Treatment of High Blood Cholesterol in Adults (Adult Treatment Panel III) final report Circulation 2002 106 3143 3421 12485966

[B23] Grundy SM Cleeman JI Daniels SR Donato KA Eckel RH Franklin BA Gordon DJ Krauss RM Savage PJ Smith SC Jr Spertus JA Fernando Costa Diagnosis and management of the metabolic syndrome: an American Heart Association/National Heart, Lung, and Blood Institute Scientific Statement Circulation 2005 112 2735 2752 10.1161/CIRCULATIONAHA.105.169404 16157765

[B24] Tan CE Ma S Wai D Chew Sk Tai ES Can we apply the national cholesterol education program adult treatment panel definition of the metabolic syndrome to Asians Diabetes Care 2004 27 1182 1186 10.2337/diacare.27.5.1182 15111542

[B25] Xu L Jiang CQ Lam TH Lin JM Yue XJ Cheng KK Liu B Jin YL Zhang WS Thomas GN The Metabolic syndrome is associated with subclinical atherosclerosis independent of insulin resistance:Guangzhou Biobank Cohort Study-CVD Clin Endocrinol 2010 73 181 188 10.1111/j.1365-2265.2009.03760.x 20039893

[B26] Li WC Hsiao KY Chen IC Chang YC Wang SH Wu KH Serum leptin is associated with cardiometabolic risk and predicts metabolic syndrome in Taiwanese adults Cardiovasc Diabetol 2011 10 36 10.1186/1475-2840-10-36 21526991 PMC3098150

[B27] Emilsson V Liu YL Cawthorne MA Morton NM Davenport M Expression of the functional leptin receptor mRNA in pancreatic islets and direct inhibitory action of leptin on insulin secretion Diabetes 1997 46 313 316 10.2337/diabetes.46.2.313 9000710

[B28] Van Gaal LF Wauters MA Mertens IL Considine RV De Leeuw ID Clinical endocrinology of human leptin Int J Obes Relat Metab Disord 1999 23 29 36 10.1038/sj.ijo.0800792 10193859

[B29] Cleland SJ Sattar N Petrie JR Forouhi NG Elliott HL Connell JM Endothelial dysfunction as a possible link between C-reactive protein levels and cardiovascular disease Clin Sci (Lond) 2000 98 531 535 10.1042/CS20000013 10781383

[B30] Heinrich PC Castell JV Andus T Interleukin-6 and the acute phase response Biochem J 1990 265 621 636 1689567 10.1042/bj2650621 PMC1133681

[B31] Mohamed-Ali V Goodrick S Rawesh A Katz DR Miles JM Yudkin JS Klein S Coppack SW Subcutaneous adipose tissue releases interleukin-6, but not tumor necrosis factor-alpha, in vivo J Clin Endocrinol Metab 1997 82 4196 4200 10.1210/jc.82.12.4196 9398739

[B32] Hotamisligil GS Arner P Caro JF Atkinson RL Spiegelman BM Increased adipose tissue expression of tumor necrosis factor-alpha in human obesity and insulin resistance J Clin Invest 1995 95 2409 2415 10.1172/JCI117936 7738205 PMC295872

[B33] Weisberg SP McCann D Desai M Rosenbaum M Leibel RL Ferrante AW Jr Obesity is associated with macrophage accumulation in adipose tissue J Clin Invest 2003 112 1796 1808 14679176 10.1172/JCI19246 PMC296995

[B34] Kirchgessner TG Uysal KT Wiesbrock SM Marino MW Hotamisligil GS Tumor necrosis factor-alpha contributes to obesity-related hyperleptinemia by regulating leptin release from adipocytes J Clin Invest 1997 100 2777 2782 10.1172/JCI119824 9389742 PMC508482

[B35] Trujillo ME Sullivan S Harten I Schneider SH Greenberg AS Fried SK Interleukin-6 regulates human adipose tissue lipid metabolism and leptin production in vitro J Clin Endocrinol Metab 2004 89 5577 5582 10.1210/jc.2004-0603 15531514

[B36] Santos-Alvarez J Goberna R Sánchez-Margalet V Human leptin stimulates proliferation and activation of human circulating monocytes Cell Immunol 1999 194 6 11 10.1006/cimm.1999.1490 10357875

[B37] Baumann H Morella KK White DW Dembski M Bailon PS Kim H Lai CF Tartaglia LA The full-length leptin receptor has signaling capabilities of interleukin 6-type cytokine receptors Proc Natl Acad Sci U S A 1996 93 8374 8378 10.1073/pnas.93.16.8374 8710878 PMC38678

[B38] Chen K Li F Li J Cai H Strom S Bisello A Kelley DE Friedman-Einat M Skibinski GA McCrory MA Szalai AJ Zhao AZ Induction of leptin resistance through direct interaction of C-reactive protein with leptin Nat Med 2006 12 425 432 10.1038/nm1372 16582918

[B39] Chen K Li F Li J Cai H Strom S Bisello A Kelley DE Friedman-Einat M Skibinski GA McCrory MA Szalai AJ Zhao AZ Induction of leptin resistance through direct interaction of C-reactive protein with leptin Nat Med 2006 12 425 432 10.1038/nm1372 16582918

[B40] Shimizu H Oh-I S Okada S Mori M Leptin resistance and obesity Endocr J 2007 54 17 2 10.1507/endocrj.KR-85 17053294

[B41] Sung KC Seo MH Rhee EJ Wilson AM Elevated fasting insulin predicts the future incidence of metabolic syndrome: a 5-year follow-up study Cardiovasc Diabetol 2011 10 108 10.1186/1475-2840-10-108 22129309 PMC3275515

[B42] Chien KL Lin HJ Lee BC Hsu HC Chen MF Prediction model for high glycated hemoglobin concentration among ethnic Chinese in Taiwan Cardiovasc Diabetol 2010 9 59 10.1186/1475-2840-9-59 20875098 PMC2955643

[B43] Farb MG Bigornia S Mott M Tanriverdi K Morin KM Freedman JE Joseph L Hess DT Apovian CM Vita JA Gokce N Reduced adipose tissue inflammation represents an intermediate cardiometabolic phenotype in obesity J Am Coll Cardiol 2011 58 232 237 10.1016/j.jacc.2011.01.051 21737012 PMC3132399

